# Features of the urban environment associated with *Aedes aegypti* abundance in high-rise public apartments in Singapore: An environmental case-control study

**DOI:** 10.1371/journal.pntd.0011075

**Published:** 2023-02-02

**Authors:** Stephanie A. Fernandez, Haoyang Sun, Borame L. Dickens, Lee Ching Ng, Alex R. Cook, Jue Tao Lim

**Affiliations:** 1 Saw Swee Hock School of Public Health, National University of Singapore and National University Health System, Singapore; 2 Environmental Health Institute, National Environmental Agency, Singapore; 3 School of Biological Sciences, Nanyang Technological University, Singapore; 4 Lee Kong Chian School of Medicine, Nanyang Technological University, Singapore; Duke-NUS GMS, SINGAPORE

## Abstract

*Aedes aegypti* abundance in residential estates is hypothesized to contribute to localised outbreaks of dengue in Singapore. Knowing the factors in the urban environment underlying high *Ae*. *aegypti* abundance could guide intervention efforts to reduce *Ae*. *aegypti* breeding and the incidence of dengue. In this study, objective data on *Ae*. *aegypti* abundance in public apartment blocks estimated by Singapore’s nationally representative Gravitrap surveillance system was obtained from the National Environmental Agency. Low and high abundance status public apartment blocks were classified based on the Gravitrap *Aegypti* Index, corresponding to the lowest and highest quartiles respectively. An environmental case-control study was conducted, wherein a blinded assessment of urban features hypothesised to form breeding habitats was conducted in 50 randomly sampled public apartment blocks with low and high abundance statuses each. Logistic regression was performed to identify features that correlated with abundance status. A multivariable logistic model was created to determine key urban features found in corridors and void decks which were predictive of the *Ae*. *aegypti* abundance status of the public apartment block. At a statistical level of significance of 0.20, the presence of gully traps [Odds Ratio (OR): 1.34, 95% Confidence Interval (CI): 1.10, 1.66], age of the public apartment block [OR: 2.23, 95% CI: 1.48, 3.60], housing price [OR: 0.33, 95% CI: 0.16, 0.61] and corridor cleanliness [OR: 0.67, 95% CI: 0.40, 1.07] were identified as important predictors of abundance status. To reduce *Ae*. *aegypti* abundance around public apartment blocks and potential onward dengue transmission, gully traps could be remodelled or replaced by other drainage types. Routine inspections of *Ae*. *aegypti* breeding should be targeted at older and low-income neighbourhoods. Campaigns for cleaner corridors should be promoted.

## Author summary

Dengue outbreaks are frequent in Singapore. Controlling the mosquito population remains the principal method in preventing dengue transmission since safe and effective pharmaceutical interventions and vaccines have not been developed. Typical vector control campaigns that strive to reduce the mosquito population urge relevant stakeholders, including residents, town councils responsible for common areas, schools and construction site contractors, to remove stagnant water from their premises. However, breeding sites have been found at close proximity to but outside of homes, including containers, plants and drains, some of which are managed by residents, others by the government. This study investigates which urban features, which had previously been found to be common breeding habitats in homes and public areas, contributed most to the *Ae*. *aegypti* abundance of residential areas. We used objective Gravitrap surveillance data to estimate *Ae*. *aegypti* abundance and an exposure assessment tool to measure the presence of urban features. Findings from the study indicate that gully traps, age of high-rise housing blocks, housing unit price and corridor cleanliness were the most predictive of *Ae*. *aegypti* abundance. Both residents and the government should be enlisted to uphold effective vector control efforts.

## Introduction

Over 180 species of mosquitoes are endemic in the equatorial city state of Singapore [1] including *Aedes aegypti*, the predominant vector of dengue viruses (DENV). Individuals bitten by a DENV-carrying mosquito may contract dengue fever, characterized by symptoms such as fever, joint pains, and skin rashes; most recover within a few days. However, a proportion of cases escalate into severe dengue haemorrhagic fever which can be life-threatening, ranging from 0.1% to 0.8% from 2012 to 2021 [2]. Dengue is endemic in Singapore, with year-round transmission, and although outbreaks commonly peak in the middle of the year, between June and September, peaks at other times of the year do occur [2,3]. The economic cost amounted to about $2 billion from 2010 to 2020 [4], with the *Wolbachia-Aedes* suppression project costing minimally $50,453 per disability-adjusted life year averted [4]. In spite of government and community efforts, a record high of 35 261 cases [2] and 32 deaths [5] were reported in 2020, potentially a by-product of measures to control the coronavirus disease 2019 pandemic [6–8]. Due to the ongoing occurrence of large outbreaks of diagnosed cases, and the falling levels of immunity in the population [9], public health measures to reduce the transmission of dengue are therefore crucial. Some current approaches include vector control measures such as fogging, premises inspections and social mobilisation to reduce sources of mosquito breeding which are augmented before and during epidemic phases [10], as well as the use of *Wolbachia* technology which has been successfully piloted to further suppress the *Ae*. *aegypti* population and is currently covering 30% of all high-rise public housing (Housing Development Board, HDB, blocks) [11,12].

Without pharmaceutical interventions [13] and vaccines with unconvincing safety profiles which commonly predispose immunological naïve individuals to higher risks of severe dengue [14], environmental control through source reduction efforts to reduce the mosquito population remains the primary mean to reduce the transmission of dengue. After a single blood meal, a female mosquito can lay up to 100 eggs at the edge on a stagnant, wet surface [15]. Ina few days, up to several months, after submerging into water, the eggs can hatch into larvae, which develop into pupae, and then finally emerge as adult mosquitoes. Common peridomestic breeding habitats identified by the NEA include containers, flower pot plates, plants (hardened soil and plant axils), plastic sheets, toilet bowls, cisterns, discarded receptacles, gully traps, covered carparks and various types of drain (roadside, scupper, covered perimeter and open perimeter) [16].

One strategy employed by Singapore’s government to monitor the *Aedes* mosquito population is the use of Gravitraps, developed by the Environmental Health Institute [17], which contain a hay infusion designed to lure gravid mosquitoes, whereupon they become stuck on an inner lining and their eggs fall through a wire mesh; newly hatched adults remain trapped under the mesh. Since 2020, almost 70,000 Gravitraps have been placed along corridors of high-rise apartment blocks and certain private landed estates [18]. Fortnightly routine checks on the Gravitraps allow public health officials to survey the mosquito population in the area by collating the number of mosquitoes trapped [19]. The Gravitrap *aegypti* index (GAI), which is the number of female adult *Ae*. *aegypti* caught divided by the number of functional Gravitraps in the area, was developed as a proxy measure of the adult mosquito population in a housing block or area. It has been assessed to be a more accurate tool to estimate dengue transmission risk than traditional indices, such as the House, Container, and Breteau Indices, since dengue is transmitted through adult female mosquitoes, whereas traditional indices measure larvae/pupae infestations [20] and are prone to underestimation since breeding sites may be cryptic [3].

While the GAI has been found to be positively correlated with areas with high breeding percentages (breeding percentage is calculated by taking the number of *Ae*. *Aegypti* positive breeding sites over *Aedes* species positive breeding sites) and cases of dengue [20], it remains unclear if there is a direct correlation between the urban features recognised as popular breeding habitats identified by the NEA and the GAI-determined *Ae*. *aegypti* abundance statuses. If the numbers and types of such urban features in the area could be shown to possess a strong predictive capacity of the abundance statuses, areas at high-risk of dengue cases could be identified and targeted for vector control efforts. This would be particularly useful during inter-epidemic periods when case counts are highly stochastic and thus may not predict risk well.

As most Gravitraps are deployed along corridors of high-rise public housing blocks [21], this study focused on investigating if the presence of urban features recognised as popular breeding habitats by the NEA in and surrounding public apartment blocks could correlate with and be used to predict the *Ae*. *aegypti* abundance statuses as determined by the GAI.

## Methods

### Study design and data measurement

The study adopted an environmental case control design using Gravitrap data from 2020 and data on the presence of urban features from December 2021 to January 2022.

Gravitrap data was obtained from the NEA who placed Gravitraps along common corridors of public apartment blocks in a ratio of about 1 Gravitrap for every 20 units, such that there were traps distributed evenly on lower, middle, and higher floors [20]. No Gravitraps were placed within the housing units. Gravitraps were checked for mosquitoes every 2 weeks. The GAI of public apartment block *b*, *G*_*bT*_, for every 2 weeks *T* was calculated based on the formula:

$$G_{bT}=\frac{\sum_{t\in T}F_{bt}}{\sum_{t\in T}N_{bt}}$$

where *F*_*bt*_ is the number of female adult Ae. aegypti caught at block *b* at visit *t* falling in the 2 weeks of interest, *T*, and *N*_*bt*_ is the number of functional Gravitraps in the same place and time.

Average GAI over the time period for each public apartment block was categorised into quartiles, and blocks with GAI in the lowest and highest quartiles were classified as having low (control) and high (case) Ae. aegypti abundance status respectively. While keeping a buffer of 100 meters around each public apartment block, a sample of 100 public apartment blocks was obtained by stratified random sampling by equal allocation from the list of low and high abundance status public apartment blocks in Singapore by one of the authors (ARC). As such, 50 public apartment blocks from each abundance status were to be assessed.

Data collection was undertaken by one of the authors (SAF), who was blinded to the abundance status. The assessment tool consisted of a standardized data entry form and rubric for grading urban features. To develop the set of urban features that were assessed in this study, we took the list of the main breeding habitats identified by the NEA as an initial basis and identified those habitats that could be assessed in public areas of the block.

Mosquitoes tend to oviposit near ground level [20,22,23], therefore urban features along the second-floor corridor, which have previously been found to capture higher levels of mosquito breeding [17], were assessed. For public apartment blocks without residential units on the second floor, with unconventional floor numbering, or where the second floor was inaccessible (such as double-storeyed maisonettes), the lowest floor with residential units that was accessible was assessed. Two categories of urban features along public apartment corridors were assessed: plants and containers. Plants and containers were assumed to belong to the unit where they were placed at the closest proximity to.

Breeding habitats present on void decks on the ground floor, or in public areas, of public apartment blocks were also assessed. The operational definition of a public area corresponding to a public apartment block was established as any area on the ground level no further than 10 meters from any physical structure of that block that was accessible to the general public. Four types of urban features in 10 randomly selected 10- by 10-meter quadrants (S1 Fig describes the selection process) in the public area of a public apartment block were assessed: gully traps, open drains, covered drains and plants. Subjective assessment of the cleanliness of both the second-floor corridor and the “void deck” on the ground floor (public housing in Singapore harks back to traditional Malay housing on stilts for air circulation) was also conducted. Table 1 provides more details on how the variables were defined and parameterised (images of certain variables are available in S5 to S10 Figs).

**Table 1:** Definitions and parameters of variables assessed.

Variable	Definition	Calculation
**Second-floor corridor**		
Plant	• Any form of vegetation with or without axils, aerial or soil type, not limited to trees, shrubs and flowers cultured in pots or trays • Plant pots containing only soil and no plant were considered plants • Multiple plants growing from one pot were considered one plant	Proportion of units with more than 5 plants
Container	Any object with the capacity to hold stagnant water, including domestic and ornamental containers, as well as empty plant pots, plates or trays (refer to S5 and S6 Figs)	Proportion of units with more than 5 containers
Corridor cleanliness	Refer to Rubric in S1 Table	Scale from 1 (very dirty) to 5 (very clean)
**Void deck/public area**		
Gully traps	Usually flat and white with numerous circular-holed cover to allow water to flow through but contain smells from the U pipe under the cover (refer to S7 Fig)	Proportion of 10 randomly selected quadrants in the corresponding public apartment block’s public area where any of the listed urban features were present
Open drains	Drains with grilled covers or no covers (refer to S8 and S9 Figs)	
Covered drains	Drains fully covered, for example, by chequered plates or stone slabs (refer to S10 Fig)	
Plants	Any form of vegetation, managed or wild	
Public cleanliness	Refer to Rubric in S1 Table	Scale from 1 (very dirty) to 5 (very clean)

After fieldwork, we obtained building age from lease commencement year data obtained from the Singapore Land Authority [24,25], and median house price per public apartment block was used as a proxy for the socioeconomic status (SES) of its residents and valued at its resale price attained through a government website (https://data.gov.sg) [26], since building age and SES [27–30] have previously been found to be correlated with mosquito abundance.

No ethics approval was required since no human research was involved.

### Statistical analyses

Means and standard deviations (SDs), stratified by abundance status, were computed for all variables. Unadjusted logistic regression models were used to assess the bivariate relationship between abundance status and each urban feature, level of cleanliness and covariates. The corresponding odds ratios (ORs), 95% confidence intervals (CIs) and Wald p-values for each variable were calculated. ORs were scaled according to the type of variable such that the quantities per unit change would be meaningful to interpret. For all proportions, ORs were rescaled such that the change in risk was based on every 10% increase in proportion. For age of public apartment block, rescaling was done for every 10 years. ORs for house price were scaled to $100,000 while for cleanliness, ORs were scaled on per score unit.

Multivariable logistic regression models were used to create prediction models of public apartment abundance status in three routes. The level of statistical significance was set to 0.05 for the first route and 0.20 [31] for the second and third routes. The first and second routes included public apartment blocks located in sites where *Wolbachia*-infected *Aedes* mosquitoes [12] were released, while the third route excluded these sites to assess the possible downward bias of public apartment blocks having high abundance status during analysis:

1^st^ route: Level of statistical significance: 0.05; *Wolbachia* sites included.

2^nd^ route: Level of statistical significance: 0.20; *Wolbachia* sites included.

3^rd^ route: Level of statistical significance: 0.20; *Wolbachia* sites excluded.

Variables found to be statistically significant in bivariate analyses were included in multivariable logistic regression models. Variables with p-values above the corresponding statistical level of significance after adjusting for confounding were dropped from the multivariable model by the backwards stepwise selection method. Another multivariable regression model, but with gully traps removed, was also generated to assess the extent of risk reduction of public apartment blocks having high abundance status by removing all gully traps. Variance inflation factors were calculated for each variable in the multivariable model to assess for collinearity.

A probability threshold of having high abundance status was determined by optimising sensitivity and specificity for each prediction model. In addition to sensitivity and specificity, area under the curve (AUC), Akaike information criterion (AIC) and Hosmer-Lemeshow tests were used to select a final prediction model.

Using the final prediction model, the predicted probabilities of having high abundance status of each public apartment block was summed to compute the change in predicted probability when proportion of public spots with gully traps was modified to the optimal value of 0.

Data analyses were performed using R version 4.1.0 [32] and RStudio version 1.4.1717 [33].

## Results

The sample sizes for first and second routes analyses were reduced to 99 as there was restricted access to the second-floor corridor of one public apartment block. The public apartment block excluded from analysis was classified with low abundance status. The third analysis route had a sample size of 89 after excluding the public apartment block with restricted access and 10 public apartment blocks located within *Wolbachia* release sites [12], leaving the sample with 44 low and 45 high abundance blocks.

Statistically significant variables from bivariate analyses at a statistical level of significance of 0.20 included proportion of public spots with gully traps, proportion of units with more than 5 plants, more than 5 containers, age of public apartment block, house price and corridor and public cleanliness. At a significance level of 0.05, significant variables include proportion of public spots with gully traps, proportion of units with more than 5 plants, more than 5 containers, age of public apartment block, house price and corridor cleanliness (Table 2). For every 10% increase in proportion of public spots with gully traps present, the odds of a public apartment block having a high abundance status were 1.28 times higher (95% CI: 1.11, 1.50). With regards to age of public apartment block, for every 10-year increase in age, the odds of having high abundance status were 2.55 times higher (95% CI: 1.80, 3.85).

**Table 2:** Unadjusted logistic regression.

Variable (scale)	Mean (SD)		OR (95% CI)	p-value
	Low abundance status	High abundance status		
Proportion of public spots with gully traps	18% (30%)	40% (30%)	1.28 (1.11, 1.50) per 10%	0.0012
Proportion of public spots with open drains	78% (21%)	81% (18%)	1.08 (0.88, 1.34) per 10%	0.46
Proportion of public spots with covered drains	70% (23%)	74% (17%)	1.10 (0.90, 1.35) per 10%	0.37
Proportion of public spots with plants	79% (19%)	77% (18%)	0.94 (0.75, 1.17) per 10%	0.59
Proportion of units with >5 plants	19% (23%)	32% (22%)	1.29 (1.07, 1.57) per 10%	0.0094
Proportion of units with >5 containers	7% (12%)	16% (19%)	1.45 (1.10, 2.02) per 10%	0.015
Age of public apartment block	20y (16y)	37y (9y)	2.55 (1.80, 3.85) per 10y	<0.0001
House price	$513 000 ($115 000)	$414 000 ($117 000)	0.46 (0.29, 0.68) per $100 000	0.0003
Corridor cleanliness (per scale unit)	3.80 (1.24)	2.98 (0.96)	0.51 (0.34, 0.75) per scale unit	0.0010
Public cleanliness (per scale unit)	3.06 (1.33)	2.66 (1.27)	0.79 (0.57, 1.07) per scale unit	0.13

SD: standard deviation; OR: odds ratio; CI: confidence interval. Means, SDs, ORs and CIs were rounded to 2 decimal places except for age of public apartment block and house price where means and SDs were rounded to the nearest whole number and thousand respectively. ORs indicate the odds of a public apartment block having high compared to low abundance status based on the corresponding variable. CIs and p values were computed based on Wald’s test.

Table 3 displays the results of multivariable logistic regression modelling. A statistical significance level of 0.05 was used to create Models 1 and 2, while that of 0.20 was used for Models 3 to 6. Variables that contributed significant risk to abundance status in the unadjusted models shown in Table 2, inclusive of *Wolbachia* release sites, were included in Models 1 and 3, while that excluding *Wolbachia* sites were included in Model 5. After applying the backward stepwise selection method, Models 2, 4 and 6 retained variables with p-values below the corresponding level of significance from Models 1, 3 and 5 respectively. Public cleanliness was removed from Model 4 despite retaining significance due to the change in direction of the coefficient in the multivariable model compared to the unadjusted model. Variance inflation factors of all variables in Models 1 to 6 were below the value of 3, demonstrating modest multi-collinearity.

**Table 3:** Multivariable logistic regression modelling.

Predictor (scale)	Route 1: a = 0.05, *Wolbachia* sites included		Route 2: a = 0.20, *Wolbachia* sites included		Route 3: a = 0.20, *Wolbachia* sites excluded	
	Model 1 OR (95% CI) [p-value]	Model 2 OR (95% CI) [p-value]	Model 3 OR (95% CI) [p-value]	Model 4 OR (95% CI) [p-value]	Model 5 OR (95% CI) [p-value]	Model 6 OR (95% CI) [p-value]
Proportion of public spots with gully traps (per 10%)	1.34 (1.09, 1.64) [0.0083]	1.32 (1.10, 1.64) [0.0053]	1.37 (1.11, 1.73) [0.0051]	1.34 (1.10, 1.66) [0.0047]	1.39 (1.12, 1.81) [0.0064]	1.37 (1.10, 1.77) [0.0079]
Proportion of units with >5 plants (per 10%)	1.14 (0.85, 1.53) [0.38]		1.14 (0.85, 1.56) [0.39]		1.22 (0.90, 1.69) [0.22]	1.28 (1.00, 1.68) [0.058]
Proportion of units with >5 containers (per 10%)	1.08 (0.73, 1.68) [0.72]		1.08 (0.71, 1.69) [0.72]		1.06 (0.69, 1.70) [0.80]	
Age of public apartment block (per 10y)	2.18 (1.45, 3.53) [0.0005]	2.38 (1.61, 3.79) [<0.0001]	2.48 (1.59, 4.25) [0.0002]	2.23 (1.48, 3.60) [0.0003]	2.49 (1.57, 4.38) [0.0004]	2.42 (1.57,4.09) [0.0002]
House price (per $100 000)	0.35 (0.17, 0.63) [0.0019]	0.32 (0.15, 0.58) [0.0006]	0.30 (0.13, 0.57) [0.0012]	0.33 (0.16, 0.61) [0.0013]	0.26 (0.11, 0.52) [0.0007]	0.266 (0.11, 0.51) [0.0006]
Corridor cleanliness (per scale unit)	0.71 (0.42, 1.18) [0.19]		0.45 (0.21, 0.88) [0.026]	0.67 (0.40, 1.07) [0.10]	0.58 (0.24, 1.25) [0.18]	
Public cleanliness (per scale unit)			1.88 (1.03, 3.70) [0.050]		1.54 (0.79, 3.35) [0.23]	

OR: odds ratio; CI: confidence interval. ORs and CIs were rounded to 2 decimal places. ORs indicate the odds of a public apartment block having high compared to low abundance status based on the corresponding variable. CIs and p values were computed based on Wald’s test.

Table 4 shows AIC, Hosmer-Lemeshow *χ*^2^ and p-values, AUC, probability thresholds, sensitivity and specificity levels for each multivariable model. Probability thresholds were selected to optimize overall accuracy as described in the methods. Models 3 and 5 show good and comparable qualities of fit of data and all six models show excellent abilities to make accurate predictions. AIC values of Models 1 to 4 will not be compared to that of Models 5 and 6 since different data was used. Of Models 1 to 4, Model 4 had the lowest AIC value of 87.53. Models 3 and 5 showed goodness-of-fit through the Hosmer-Lemeshow test, with Model 4 on the borderline with a p-value of 0.05 and Models 2 and 6 with a p-value below 0.05, suggesting some mischaracterisation of the risk model. AUC of all six models were high at values equal to or above 0.90. At the corresponding probability threshold, sensitivity and specificity were at least 0.80 for all models, with Model 4 having the highest sensitivity at 0.90 and Model 1 having the highest specificity value at 0.90.

**Table 4:** Comparison of multivariable models.

Model	Route 1: a = 0.05, *Wolbachia* sites included		Route 2: a = 0.20, *Wolbachia* sites included		Route 3: a = 0.20, *Wolbachia* sites excluded	
	Model 1	Model 2	Model 3	Model 4	Model 5	Model 6
AIC	89.90	88.30	87.61	87.53	78.97	75.14
Hosmer-Lemeshow goodness-of-fit (10 quantiles)	$\chi_{8}^{2}=14.3$ p = 0.07	$\chi_{8}^{2}=16.3$ p = 0.04	$\chi_{8}^{2}=11.2$ p = 0.19	$\chi_{8}^{2}=15.6$ p = 0.05	$\chi_{8}^{2}=12.9$ p = 0.12	$\chi_{8}^{2}=20.9$ p = 0.007
AUC	0.91	0.90	0.92	0.91	0.93	0.92
Probability threshold	0.57	0.54	0.56	0.46	0.55	0.54
Sensitivity	0.82	0.82	0.86	0.90	0.87	0.88
Specificity	0.90	0.88	0.88	0.82	0.89	0.86

AIC: Akaike’s Information Criterion; AUC: Area under the receiver operating characteristics curve.

**Fig 1:**
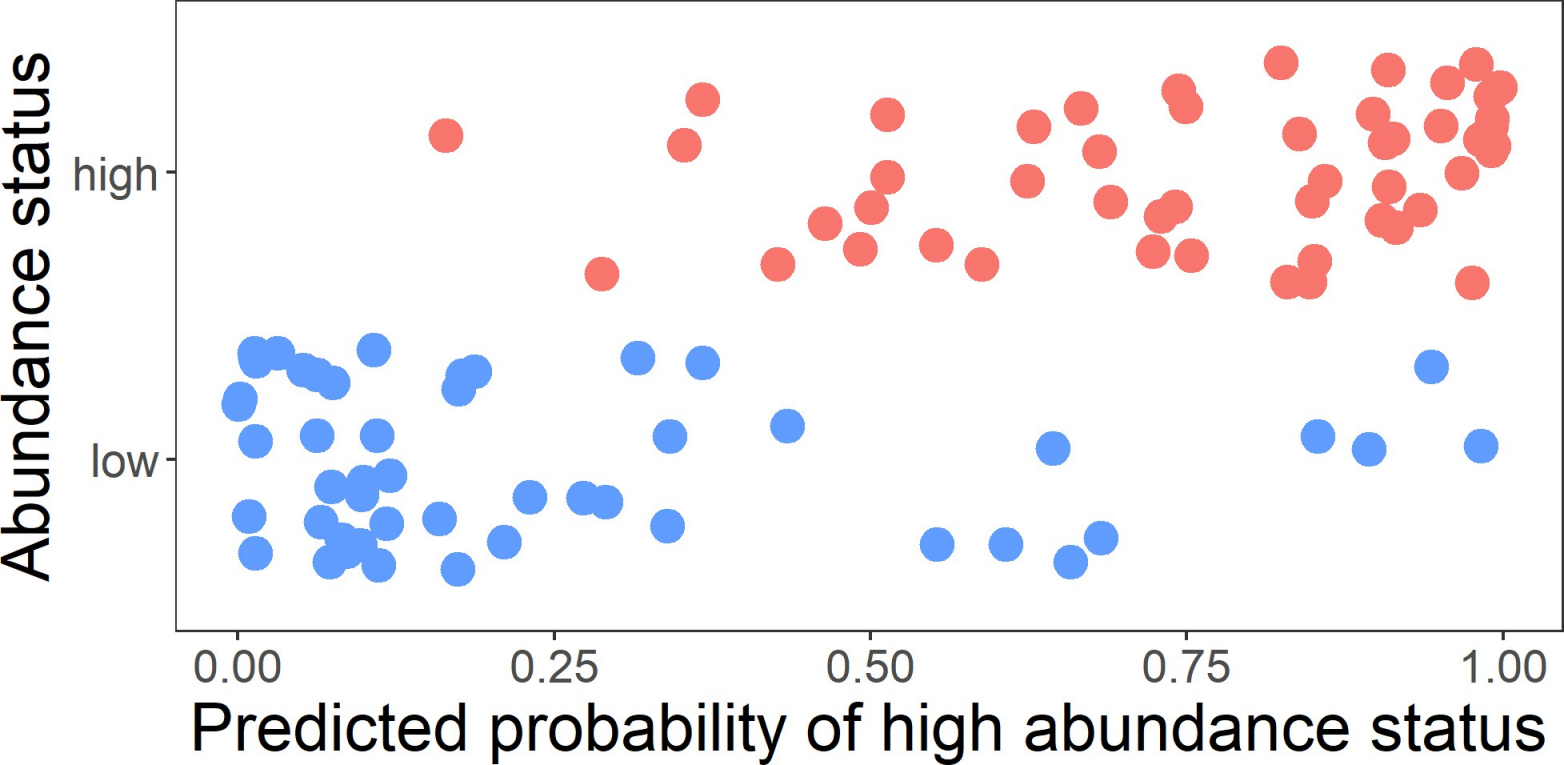
Scatter plot of abundance status against predicted probabilities of having high abundance status based on the final prediction model.

Based on having the highest sensitivity value (over specificity), Model 4 was selected as the final prediction model. Fig 1 shows the predicted probabilities of public apartment blocks having high abundance status stratified by actual abundance status using Model 4. Overlaps of predicted probabilities between high and low abundance status public apartment blocks were scarce, indicating that the final prediction model has a decent level of accuracy in distinguishing between high and low abundance status public apartment blocks.

Based on the final prediction model and the assumption that the relationship between the presence of gully traps and abundance status was causal, whereby more gully traps present would cause an increased likelihood of the apartment block to have a high abundance status, the predicted proportion of public apartment blocks in the sample with high abundance status would fall from 50 to 38 (by 23.9%; range: 15.1%—33.4%) if all gully traps were removed.

## Discussion

This study investigated the relationship between the presence of features of the urban environment in public areas of public housing estates in Singapore and *Ae*. *aegypti* abundance as estimated by the GAI. With the exception of plants in public areas, the increased presence of all types of urban features studied was associated with higher risks of high *Ae*. *aegypti* abundance. The final prediction model—which was selected to favour sensitivity over specificity as the cost of wrongly classifying a low-risk public apartment block with high *Ae*. *aegypti* abundance to residents’ health is likely to be scant—indicated that four key factors predicted public apartment block abundance status: the presence of gully traps, age of public apartment block, house price, and corridor cleanliness. Importantly, gully traps and corridor cleanliness are modifiable and thus could inform vector control to target these modifiable factors.

The release of *Wolbachia-Aedes* mosquito has been shown to suppress the dengue mosquito population by 70% to 80% in Yishun and Tampines estates [11]. Exclusion of these sites indicated that presence of gully traps, house price and age of public apartment block remained as key predictors, while corridor cleanliness was no longer important and proportion of units with more than 5 plants gained predictive strength. The model accounting for *Wolbachia* sites was comparable to the final prediction model. However, as most of Singapore has not been exposed to *Wolbachia-Aedes* mosquitoes, the final prediction model should take precedence.

Gully traps are common in public areas along the perimeter of public apartment blocks and at the water tap in the ground-floor void deck. Water stagnation in gully traps is possible below the gully frame and may be accessible to mosquitoes through traditional circular-holed gully covers. If the sampled sites were representative of all public housing in Singapore (rather than upper and lower quartiles), and the relationships causal, removing gully traps could reduce the risk of high mosquito abundance by 23.9% in the abundance-quartiles studied. Further investigation of designs that are less suitable for mosquito breeding, therefore, should be considered, such as incorporating anti-mosquito valves.

Results from this study showed that older public apartment blocks were associated with high *Ae*. *aegypti* abundance. Infrastructural degradation through cracks in concrete and accumulation of more yard debris, such as in ledges, in older public apartment blocks provide opportunities for mosquito breeding [34] and are largely inevitable, therefore current younger public apartment blocks are likely to be at high risk for mosquito breeding in the future. However, building age could also be associated with other aspects of the built environment since younger buildings are commonly situated within modern built environments, and we hypothesise that such newer built environments are engineered to hold less stagnant water on the ground. Water ponding has been of increasing concern in Singapore due to climate change and increased urbanisation [35] and to reduce the occurrences of water ponding, more recent housing developments could have been designed to have better stormwater management. Underground detention tanks, vegetated bioretention swales and basins control and channel surface runoff away from urban areas more effectively [36], thereby reducing water stagnation and breeding potential. As vegetation is a component of the modern urban strategy that could manage water ponding, this could explain why results of this study showed that presence of plants in public spots were similar in both low and high abundance status public apartment blocks. However, it is important to manage vegetation in bioretention swales and basins, and in roof gardens which are common in newer public apartment blocks, to prevent soil hardening and accumulation of plant debris which could subsequently increase *Ae*. *aegypti* abundance in the long-term. In addition, *Ae*. *Albopictus*, a poorer vector of dengue compared to *Ae*. *Aegypti* but having a preference for vegetation for oviposition sites [37], could thrive and have a heavier influence on the spread of dengue in the future if vegetation in public apartment blocks is not managed.

House price was used as a proxy for SES of residents in public apartment blocks. We found a negative correlation between SES and *Ae*. *aegypti* abundance status, which may potentially be due to differences in air-conditioning and natural ventilation used to cool apartments down. The use of air-conditioning and closing windows also decreases humidity and temperature within homes, thereby reducing the likelihood of mosquito breeding and abundance. Regardless of whether it is this or another factor at play, the study suggests that amongst public housing, inspection for breeding sites and breeding prevention measures should be targeted at lower income neighbourhoods.

Lower subjective ratings of corridor cleanliness were associated with increased risk of having high *Ae*. *aegypti* abundance. This finding is expected as wetter corridors cluttered with objects have more breeding potential. Since corridor cleanliness is influenced by public apartment block residents, more effort can be put into promotional efforts to educate the community through Do the Mozzie Wipeout. Other current measures include members of the NEA and Inter-Agency Dengue Task Force working together to remove potential mosquito breeding habitats through the Intensive Source Reduction Exercise [38]. Future studies should monitor and evaluate these interventions for their efficacy in keeping corridor cleanliness and its correlation with *Ae*. *aegypti* abundance and incidence of dengue.

Several limitations of this study should be acknowledged. This study adopted a case-control design, wherein the exposure data on urban features present in public apartment blocks were collected in 2021 and 2022, after the outcome data on GAI/ *Ae*. *aegypti* abundance of public apartment blocks in 2020. However, this is an environmental study, therefore urban features in void decks such as drains and plants were unlikely to have changed in 1 or 2 years. On the other hand, we acknowledge that residents could have modified the placement of objects or plants outside their units, since dengue cases were at a record high in 2020 [4] which could have influenced residents to keep fewer potted plants or containers along the corridors in 2021, biasing the results of this study towards the null. Other driving forces of *Ae*. *aegypti* abundance, such as weather and presence of construction sites and vegetation in 2020, were not accounted for. The study only considered high-rise public housing, therefore extrapolation of the study results to other housing types such as houses or condominiums in Singapore or overseas is not recommended. Another limitation is that features within homes were not assessed but could have influenced the *Ae*. *aegypti* abundance of the public apartment blocks. Nevertheless, the sensitivity and specificity of the final prediction model were high, at 90% and 82%, respectively, therefore any improvement in accuracy of the model may be minimal. However, the causal relationship of peri-domestic urban features and *Ae*. *aegypti* abundance remains questionable since the former could be correlated with within-home features that could be the true causal factor for the abundance status. Additionally, none of the prediction models were validated as withholding data for cross-validation was not ideal due to the low sample size of the study. Future studies with more resources could assess more high-rise apartment blocks to address this limitation.

Despite these limitations, this study shows that it is possible to develop a prediction model for public apartment block *Ae*. *aegypti* abundance status based on presence of urban features recognised to be potential mosquito breeding habitats with high accuracy. The developed model could be useful in stratifying housing estates for targeted control, and suggests two factors—the presence of gully traps and corridor cleanliness—that could be modified to potentially reduce the risk of dengue transmission in public housing estates in Singapore.

## Supporting information

S1 Dataset Dataset used and analysed for this study. Data collected for all 100 blocks assessed includes total number of units, number of units with more than 5 plants, number of units with more than 5 containers, corridor and public cleanliness rating, number of times out of the 10 public spots assessed that gully traps, open and covered drains and plants were present, median house price, year built and abundance status.
(XLSX)

S1 Table Rubrics for general upkeep assessment of HDB blocks. (DOCX)

S1 Fig Simplified diagram of the modified Google maps image of a public apartment block surveyed. The procedure for random selection of the 10 quadrants was as follows: A Google maps image (shown as a simplified diagram) with a view of the public apartment block of interest (grey block) at a 90° angle from the ground was taken. Gridlines were drawn over the image at intervals corresponding to the number of pixels representing 10 meters. As a result, each grid cell would represent a 10- by 10-meter quadrant. Grid cells located outside the operational definition of a block were excluded from further analysis. Out of the remaining grid cells, 10 were randomly selected for assessment of the presence of the four specified urban features. Magenta grids represent areas outside of the operational definition of the area corresponding to a public apartment block. Lighter blue grids indicate randomly selected quadrants assessed for presence of urban features.
(TIF)

S2 Fig A high-rise public apartment block in Singapore. Certain blocks may have fewer or more floors than what is shown in the image.
(TIF)

S3 Fig The corridor of one level of a high-rise public apartment block. Residents may place objects or grow plants outside their units.
(TIF)

S4 Fig The void deck of a high-rise public apartment block. Void decks are on the ground level of a block and are typically sheltered spaces with seating areas.
(TIF)

S5 Fig Example 1 of containers found along corridors of an apartment block. The containers shown in the image were not placed along the corridors but are examples of containers typically placed at corridors. Empty plant pots were considered containers.
(TIF)

S6 Fig Example 2 of containers found along corridors of an apartment block. The containers shown in the image were not placed along the corridors but are examples of containers typically placed at corridors. Plastic pails were commonly found along corridors.
(TIF)

S7 Fig Gully trap. (TIF)

S8 Fig An open perimeter drain, or open drain. (TIF)

S9 Fig A grilled drain, or open drain. (TIF)

S10 Fig A drain covered by a plate, or covered drain. (TIF)
