# Supplementary material for: Features of the urban environment associated with Aedes aegypti abundance in high-rise public apartments in Singapore: An environmental case-control study
Source: PLoS Negl Trop Dis. 2023 Feb 2;17(2):e0011075. doi: 10.1371/journal.pntd.0011075 (PMC9928025; doi:10.1371/journal.pntd.0011075)
Supplement: S1 Table — (DOCX) [file pntd.0011075.s002.docx]

| **Score** | **Characteristics** | |
| --- | --- | --- |
|  | **Void deck/surroundings** | **Corridor** |
| 1 – Very dirty  2 – Quite dirty | - Bins are overfilled. - A lot of trash found and appear to have been left for quite a long time. - Facilities are run-down. | - Cluttered with objects that are potential breeding habitats or already breeding mosquitoes and covered in dirt. |
| 3 – Neutral | - Bins are functional. - Trash found outside bins but they do not seem to have been left out for very long. - Facilities are moderately clean. | - Some efforts to maintain cleanliness but more can be done to reduce risk of breeding mosquitoes. Eg. Containers are clean but not flipped over. |
| 4 – Quite clean  5 – Very clean | - Bins are well-maintained. - No trash outside of bins in sight. - Facilities are clean. | - Corridor is well-maintained. - No potential breeding habitats in sight, or if present, are clean and managed well (containers flipped over). |

**S1 Table: Rubrics for general upkeep assessment of HDB blocks**
