# Supplementary figures and images for: Features of the urban environment associated with Aedes aegypti abundance in high-rise public apartments in Singapore: An environmental case-control study
Source: PLoS Negl Trop Dis. 2023 Feb 2;17(2):e0011075. doi: 10.1371/journal.pntd.0011075 (PMC9928025; doi:10.1371/journal.pntd.0011075)

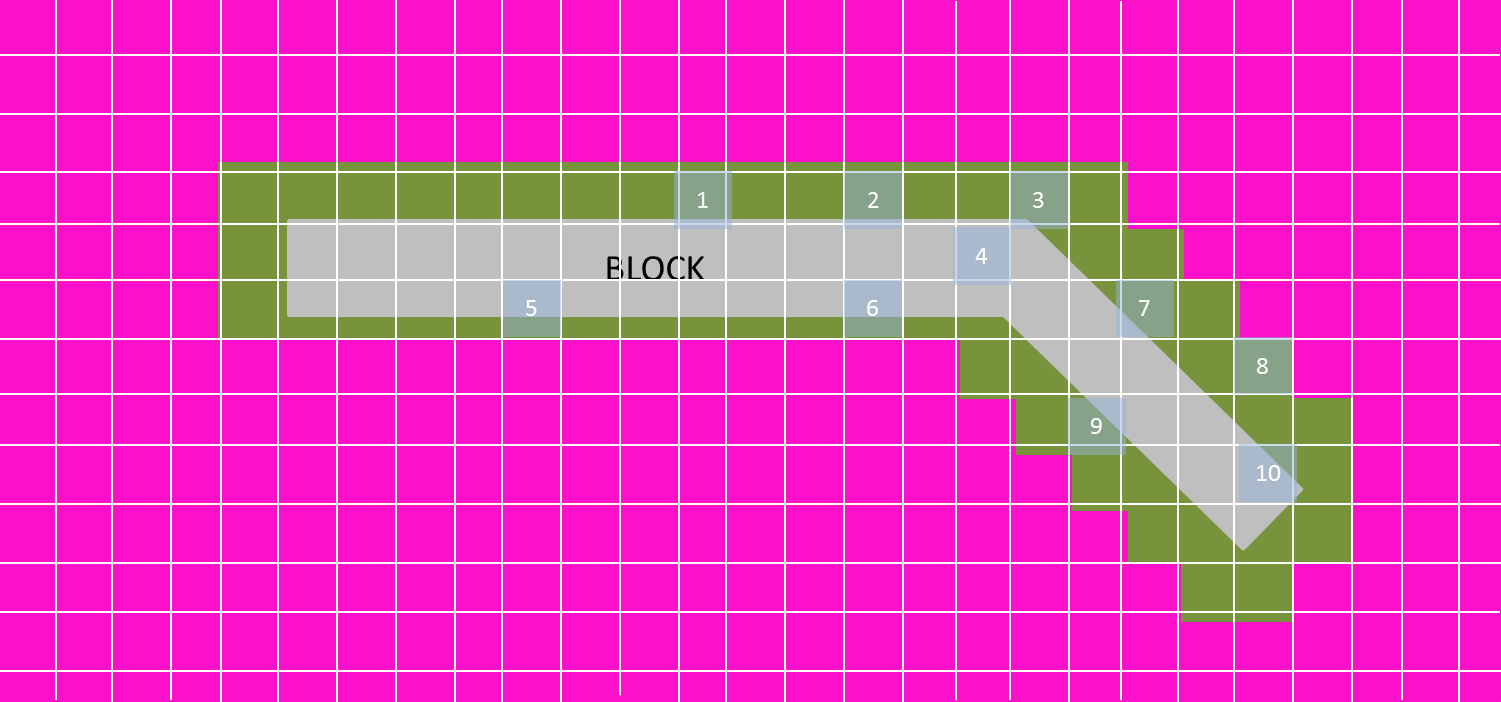

Supplement: S1 Fig — The procedure for random selection of the 10 quadrants was as follows: A Google maps image (shown as a simplified diagram) with a view of the public apartment block of interest (grey block) at a 90° angle from the ground was taken. Gridlines were drawn over the image at intervals corresponding to the number of pixels representing 10 meters. As a result, each grid cell would represent a 10- by 10-meter quadrant. Grid cells located outside the operational definition of a block were excluded from further analysis. Out of the remaining grid cells, 10 were randomly selected for assessment of the presence of the four specified urban features. Magenta grids represent areas outside of the operational definition of the area corresponding to a public apartment block. Lighter blue grids indicate randomly selected quadrants assessed for presence of urban features. (TIF) [file pntd.0011075.s003.tif]

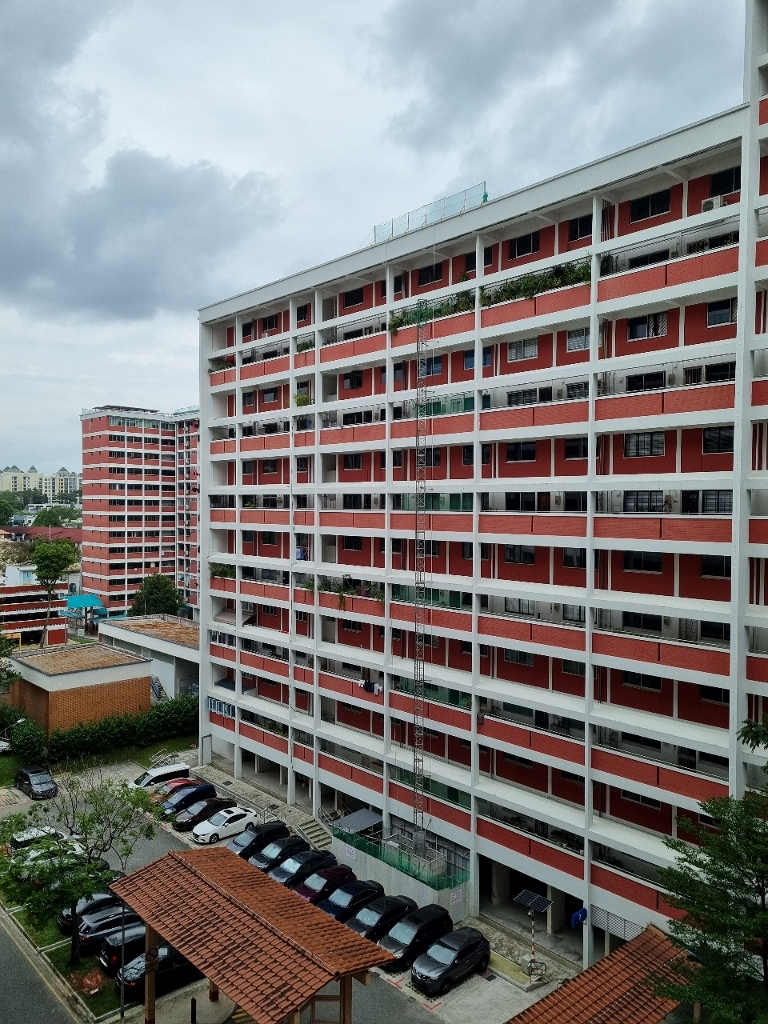

Supplement: S2 Fig — Certain blocks may have fewer or more floors than what is shown in the image. (TIF) [file pntd.0011075.s004.tif]

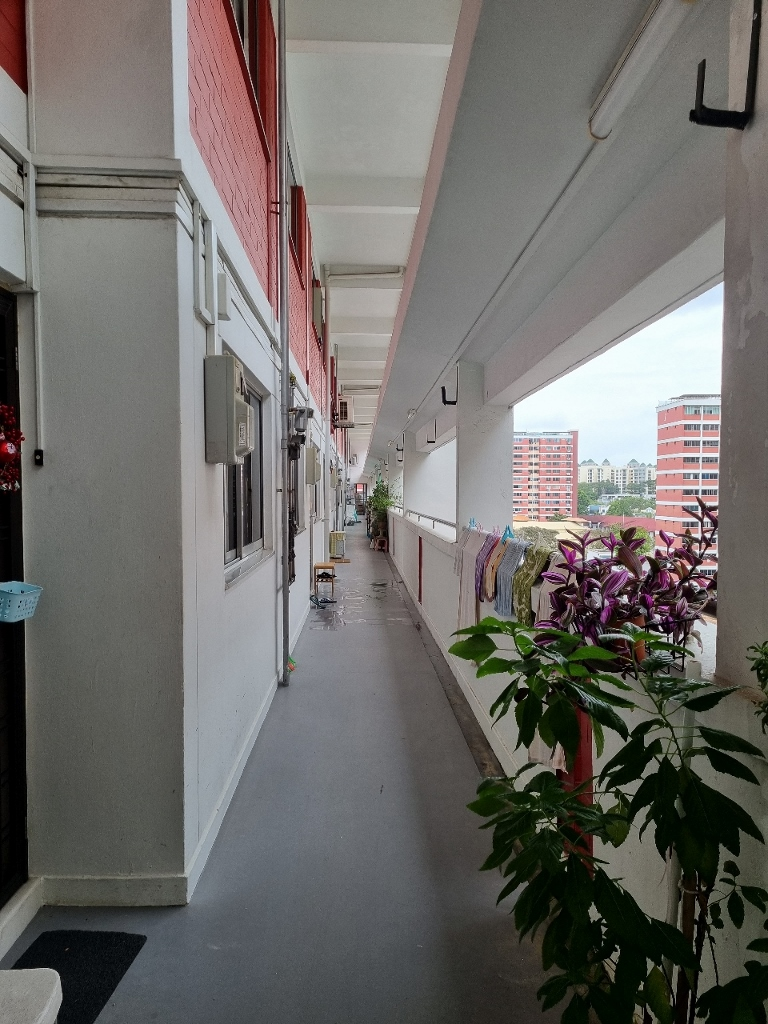

Supplement: S3 Fig — Residents may place objects or grow plants outside their units. (TIF) [file pntd.0011075.s005.tif]

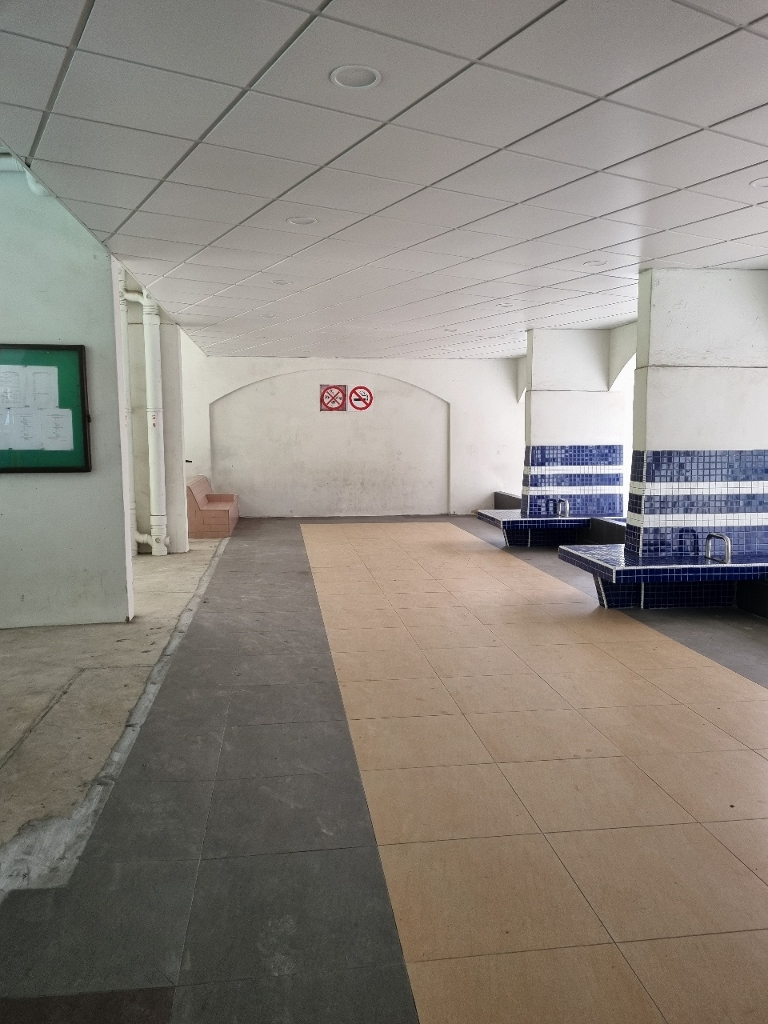

Supplement: S4 Fig — Void decks are on the ground level of a block and are typically sheltered spaces with seating areas. (TIF) [file pntd.0011075.s006.tif]

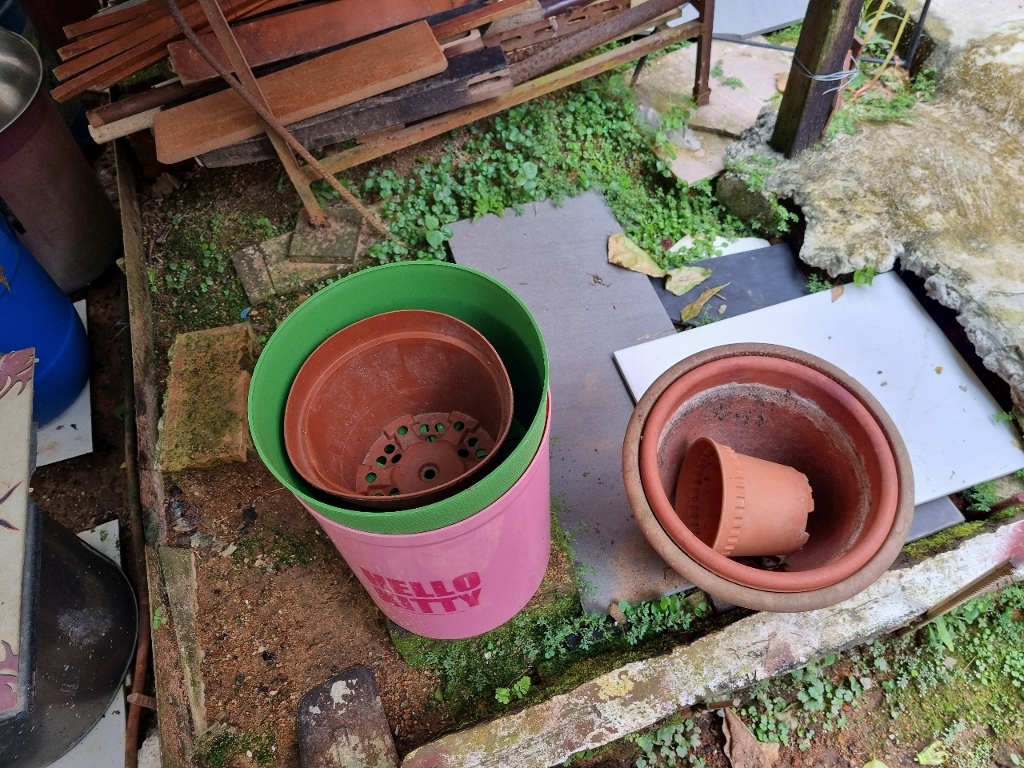

Supplement: S5 Fig — The containers shown in the image were not placed along the corridors but are examples of containers typically placed at corridors. Empty plant pots were considered containers. (TIF) [file pntd.0011075.s007.tif]

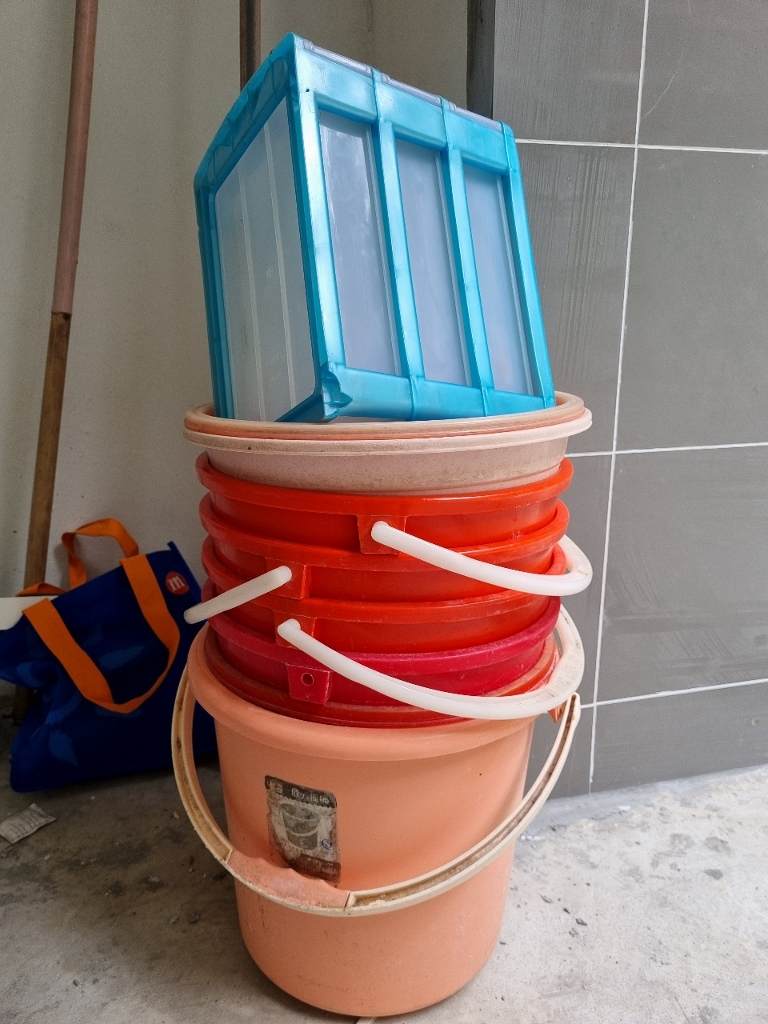

Supplement: S6 Fig — The containers shown in the image were not placed along the corridors but are examples of containers typically placed at corridors. Plastic pails were commonly found along corridors. (TIF) [file pntd.0011075.s008.tif]

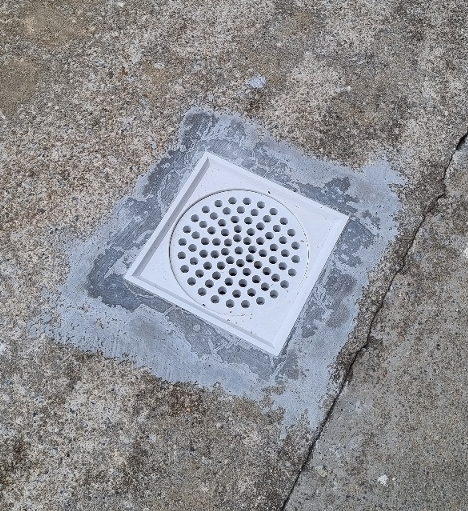

Supplement: S7 Fig — (TIF) [file pntd.0011075.s009.tif]

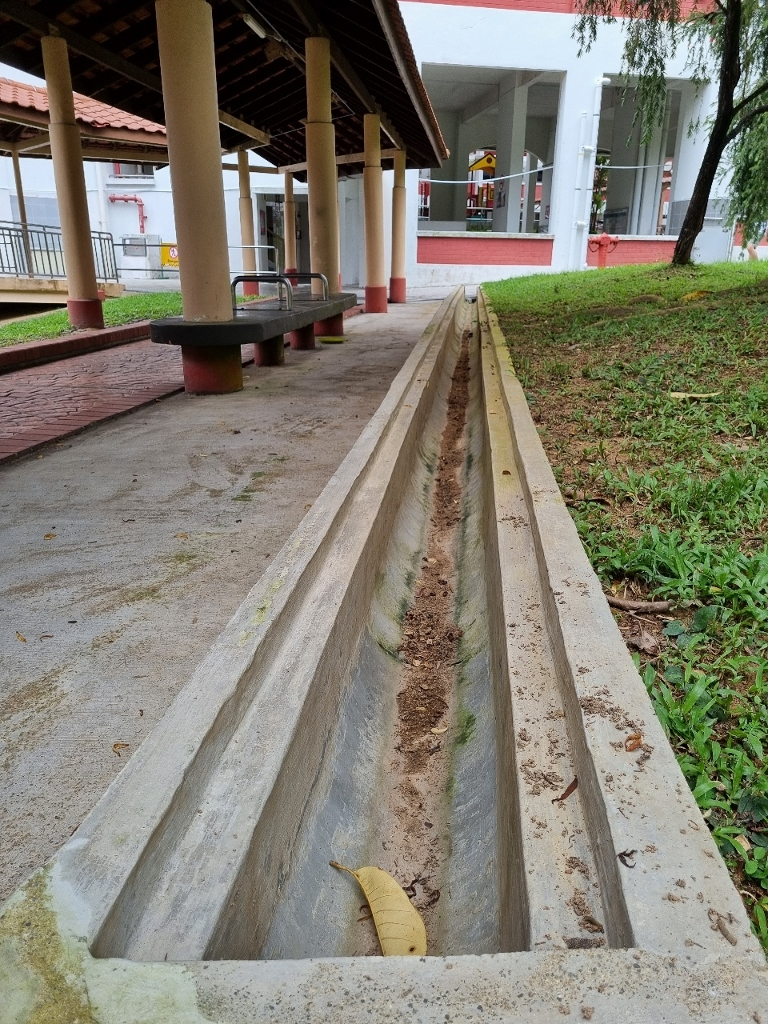

Supplement: S8 Fig — (TIF) [file pntd.0011075.s010.tif]

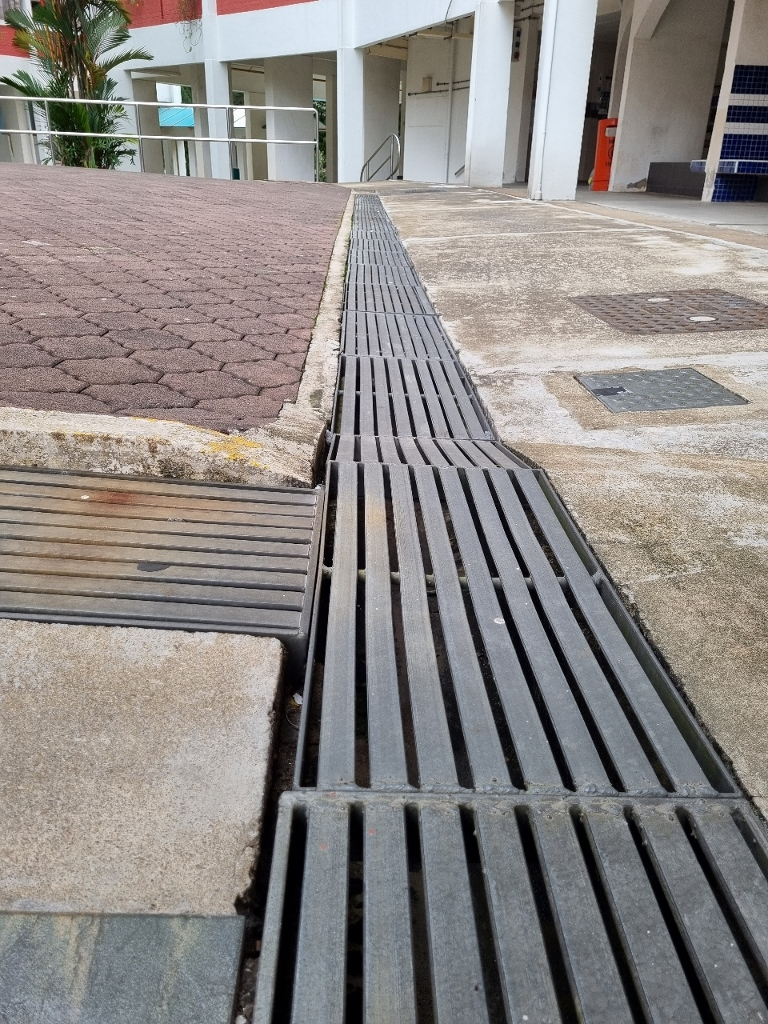

Supplement: S9 Fig — (TIF) [file pntd.0011075.s011.tif]

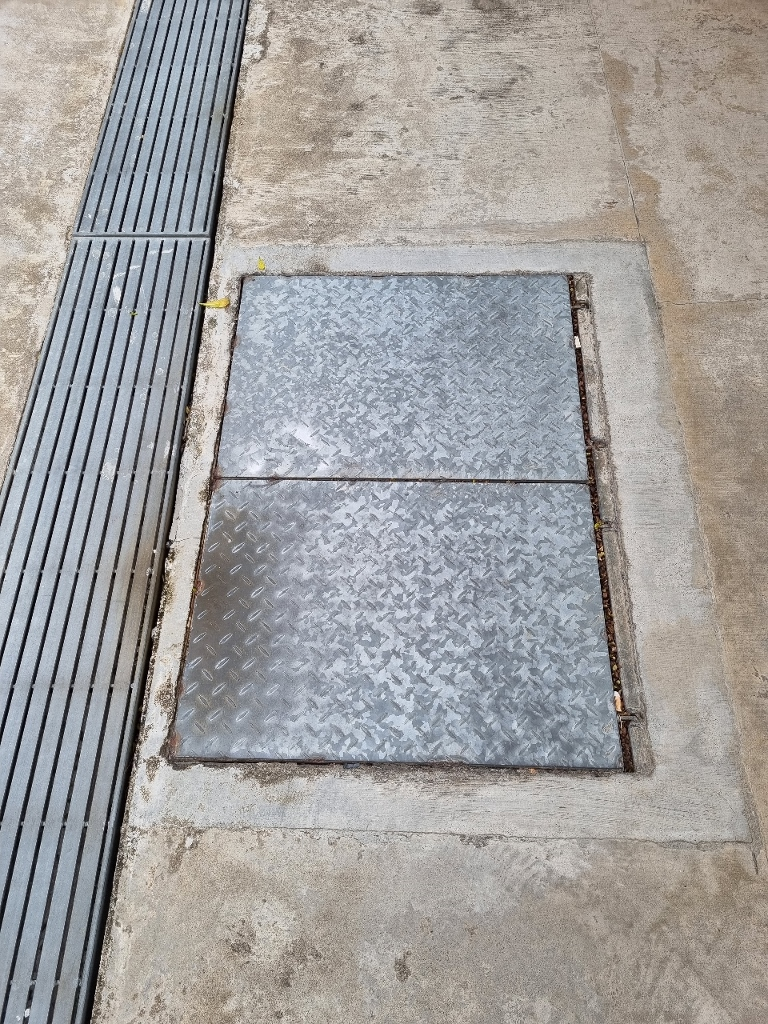

Supplement: S10 Fig — (TIF) [file pntd.0011075.s012.tif]
